# A new species of *Procamallanus* Baylis, 1923 (Nematoda, Camallanidae) from *Astronotusocellatus* (Agassiz, 1831) (Perciformes, Cichlidae) in Brazil

**DOI:** 10.3897/zookeys.790.24745

**Published:** 2018-10-15

**Authors:** Raul Henrique da Silva Pinheiro, Francisco Tiago de Vasconcelos Melo, Scott Monks, Jeannie Nascimento dos Santos, Elane Guerreiro Giese

**Affiliations:** 1 Programa de Pós-Graduação em Biologia de Agentes Infecciosos e Parasitários – Instituto de Ciências Biológicas, Universidade Federal do Pará, Belém, Pará 66075-110, Brazil; 2 Laboratório de Histologia e Embriologia Animal – Instituto da Saúde e Produção Animal – Universidade Federal Rural da Amazônia – UFRA, Belém, Pará, 66.077-830, Brazil; 3 Laboratório de Biologia Celular e Helmintologia ‘‘Profa Dra Reinalda Marisa Lanfredi’’ Instituto de Ciências Biológicas, Universidade Federal do Pará, Belém, Pará 66075-110, Brazil; 4 Laboratorio de Morfología Animal, Centro de Investigaciones Biológicas, Universidad Autónoma del Estado de Hidalgo, Pachuca, Hidalgo, 42001, México

**Keywords:** Amazon, fish, helminth, nematode

## Abstract

A new species of *Procamallanus* Baylis, 1923 was found as a parasite of the fish *Astronotusocellatus* (Agassiz, 1831) from a lake in the Jardim Botânico Bosque Rodrigues Alves, Belém, Brazil. *Procamallanusspiculastriatus***sp. n.** has a smooth buccal capsule and a well-developed basal ring that is armed with four sclerotized tooth-like structures. The male of the new species is similar to the two species that are known from Brazilian fish, *P.peraccuratus* Pinto, Fábio, Noronha & Rolas, 1976, and *P.annipetterae* Kohn & Fernandes, 1988, by the absence of the gubernaculum. It differs from these two by the morphology of the buccal capsule, the number are arrangement of the caudal papillae in males, the size and morphology of the spicules and the shape of the tail of both sexes. *Procamallanusspiculastriatus***sp. n.** is the third species discovered in fish from Brazil. This finding extends the geographical distribution of the genus into the Brazilian Amazon.

## Introduction

The genus *Astronotus* is comprised of two species, *A.crassipinnis* (Heckel, 1840) and *A.ocellatus* (Agassiz, 1831) (Perciformes: Cichlidae) (Kullander 2003; Froese and Pauly 2017). *Astronotusocellatus* (known as Acaraú-açu; Apaiari; Oscar) is popular among aquarists, but it is not as popular for aquaculture because of its slow growth rate. Early attempts at cultivation were encouraged by the government in the early 1970s, but they were not successful they only succeeded in the introduction of the species into almost all of Brazil (Fontenele and Nepomuceno 1983). Because it is an introduced species, there has been little interest on studies of its parasites; to date, only three studies (Azevedo et al. 2007; Malta 1984; Neves et al. 2013) have included this species of fish.

Nematodes of the genus *Procamallanus* Baylis, 1923 (Camallanida, Procamallaninae) are predominately parasites of freshwater fish that are distributed over several zoogeographical regions (Moravec 1998; Hoffman 1999). Members of the genus are easily recognized by the presence of a buccal capsule that is strongly sclerotized, but without ridges (Baylis 1923; Moravec and Scholz 1991; Moravec and Thatcher 1997). Despite the importance of introduced species of fish as mechanisms of co-introduction of parasites into native populations (Bautista-Hernández et al. 2014), helminths of most of introduced species have not been studied. As part of an ongoing study of the helminths of vertebrates of eastern Brazil, samples of *A.ocellatus* were collected and necropsied. Procamallanin nematodes were found as parasites of these fish, but they could not be assigned to a known species; therefore, the new species is described herein.

## Materials and methods

Forty specimens of *A.ocellatus* were collected from Iara Lake, at the Jardim Botânico Bosque Rodrigues Alves (1°25'49"S, 48°27'22"W), located in an urban area of the city of Belém, state of Pará, eastern Brazilian Amazon. Fish were collected during March to July 2015 with the aid of a casting net. Fish were transported alive to the Laboratório de Biologia Celular e Helmintologia “Profa. Dra. Reinalda Marisa Lanfredi”, Instituto de Ciências Biológicas, Universidade Federal do Pará-UFPA, for necropsy. Nematodes were collected, washed in phosphate-buffered saline, fixed in AFA solution (93 parts 70% ethanol, 5 parts formalin, and 2 parts glacial acetic acid) (Pritchard and Kruse 1982), and stored in 70% ethanol. For light microscopy, helminths were cleared in Amman’s lactophenol solution (Giese et al. 2009) and examined under an Olympus BX41 microscope with a drawing tube. Measurements are given in micrometers unless otherwise noted and are presented as the range (minimum and maximum values) followed by the mean in parentheses. For scanning electron microscopy (SEM), helminths were washed in phosphate-buffered saline with a pH of 7.0 (Sodium Phosphate Monobasic 3.12 g, Sodium Phosphate Dibasic 2.83 g, and 17 g Sodium Chloride in 200 ml of distilled water), post-fixed in 1% osmium tetroxide, dehydrated to the critical point using CO_2_, coated with gold+palladium, and studied using a scanning electron microscope (VEGA 3 LMU/TESCAN) in the Laboratório de Histologia e Embriologia Animal – Instituto da Saúde e Produção Animal – Universidade Federal Rural da Amazônia – UFRA, campus Belém, state of Pará, Brazil. Type material was deposited in the Coleção de Invertebrados of the Museu Paraense Emílio Goeldi (MPEG), Belém, Pará, Brazil. Other material examined included specimens of *Procamallanusperaccuratus* Pinto, Fábio, Noronha & Rolas, 1976, Coleção Helmintologica do Instituto Oswaldo Cruz (CHIOC): females-16.747A-C, 16.759A-C, 31.082A, 31.083A-C; males-16.753B-D, 16.757A-B, 16.769A-C, 16.773B, 31.083A-B); *Spirocamallanusinopinatus* Travassos, Artigas & Pereira, 1928, CHIOC 31.315A-B, CHIOC 31.323A-B, CHIOC 31.324, CHIOC 31.325A-B, CHIOC 31.326A-B, CHIOC 31.327, CHIOC 31.328 and CHIOC 31.329; *S.rarus* Travassos, Artigas & Pereira, 1928, CHIOC 31.027A-B, CHIOC 31.328A-C; *S.pexatus* Pinto, Fabio, Noronha & Rolas, 1974, CHIOC 31.086A-D, CHIOC 31.087, CHIOC 31.088A-B, CHIOC 31.089A-B and 32.504A-B; *S.paraensis* Pinto, Fabio, Noronha & Rolas, 1976, CHIOC 31.342A-E; and *S.saofranciscensis* Moreira, Oliveira & Costa, 1994, CHIOC 37.857, CHIOC 37.858 and CHIOC 38.042.

## Systematics

### Family Camallanidae Railliet & Henry, 1915

#### Subfamily Camallaninae Railliet & Henry, 1915

##### Genus *Procamallanus* Baylis, 1923

###### Procamallanus spiculastriatus sp. n.

Taxon classificationAnimaliaCamallanidaCamallanidae

http://zoobank.org/AD83ABF3-9B63-455C-8AC6-3B3795CD6156

Figures 1 , 2 , 3

####### Material.

**Type specimens.** Holotype male (MPEG 195), allotype female (MPEG 196), and four paratypes (MPEG 197; MPEG 198; MPEG 199; MPEG 200) were deposited in the Coleção de Invertebrados of the Museu Paraense Emílio Goeldi (MPEG), Belém, Pará, Brazil.

####### Type host.

*Astronotusocellatus* (Agassiz) (Perciformes: Cichlidae). Average length = 24.7±2.6 cm; average weight = 331.8±96.3 g.

####### Type locality.

Iará Lake, Jardim Botânico Bosque Rodrigues Alves (1°25'49"S, 48°27'22"W), Belém, Pará, Amazon Biome, Brazil.

####### Site in host.

Mid-intestine.

####### Host-parasite data.

Prevalence 55% (22 infected, 40 examined); Mean intensity = 14.8; Mean abundance = 8.5; Range = 1–59.

####### Etymology.

The species name refers to the unique morphology of the spicules, which membranous alae that are supported by rays, giving them a striated appearance.

####### Description.

**Figure 1.**
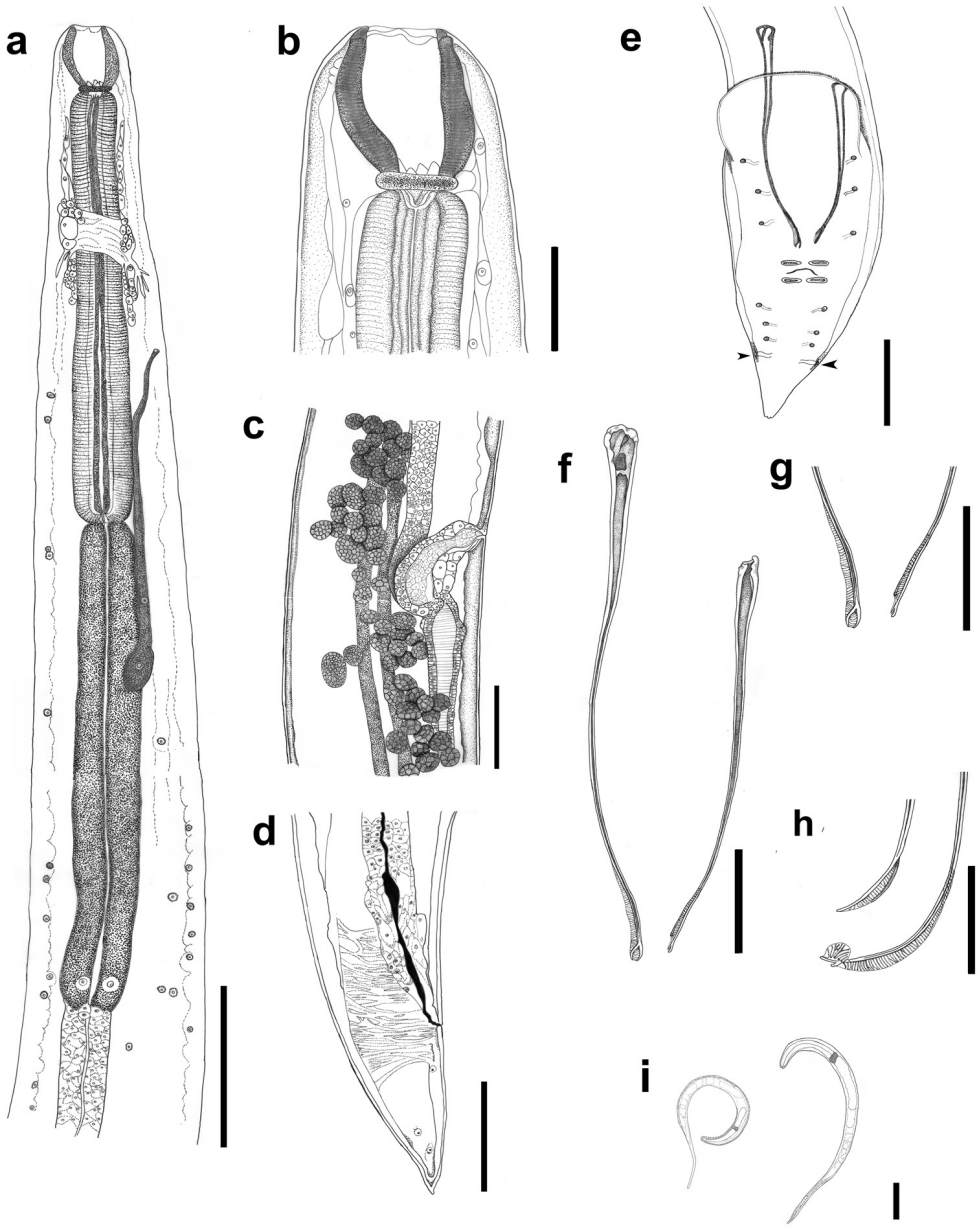
*Procamallanusspiculastriatus* sp. n., line drawings **a** adult male, anterior part of the body, lateral view **b** buccal capsule, lateral view **c** vulvar opening and vagina **d** tail of a female worm, lateral view **e** posterior end of a male worm and phasmids (arrowheads), ventral view **f** spicules, ventral view **g** distal part of spicules, ventral view **h** distal end of spicules, lateral view **i** larvae. Scale bars: 150 µm (**a**); 40 µm (**b**); 100 µm (**c, d**); 20 µm (**e**); **f**, 50 µm; 50 µm (**g, h, i**).

**Figure 2.**
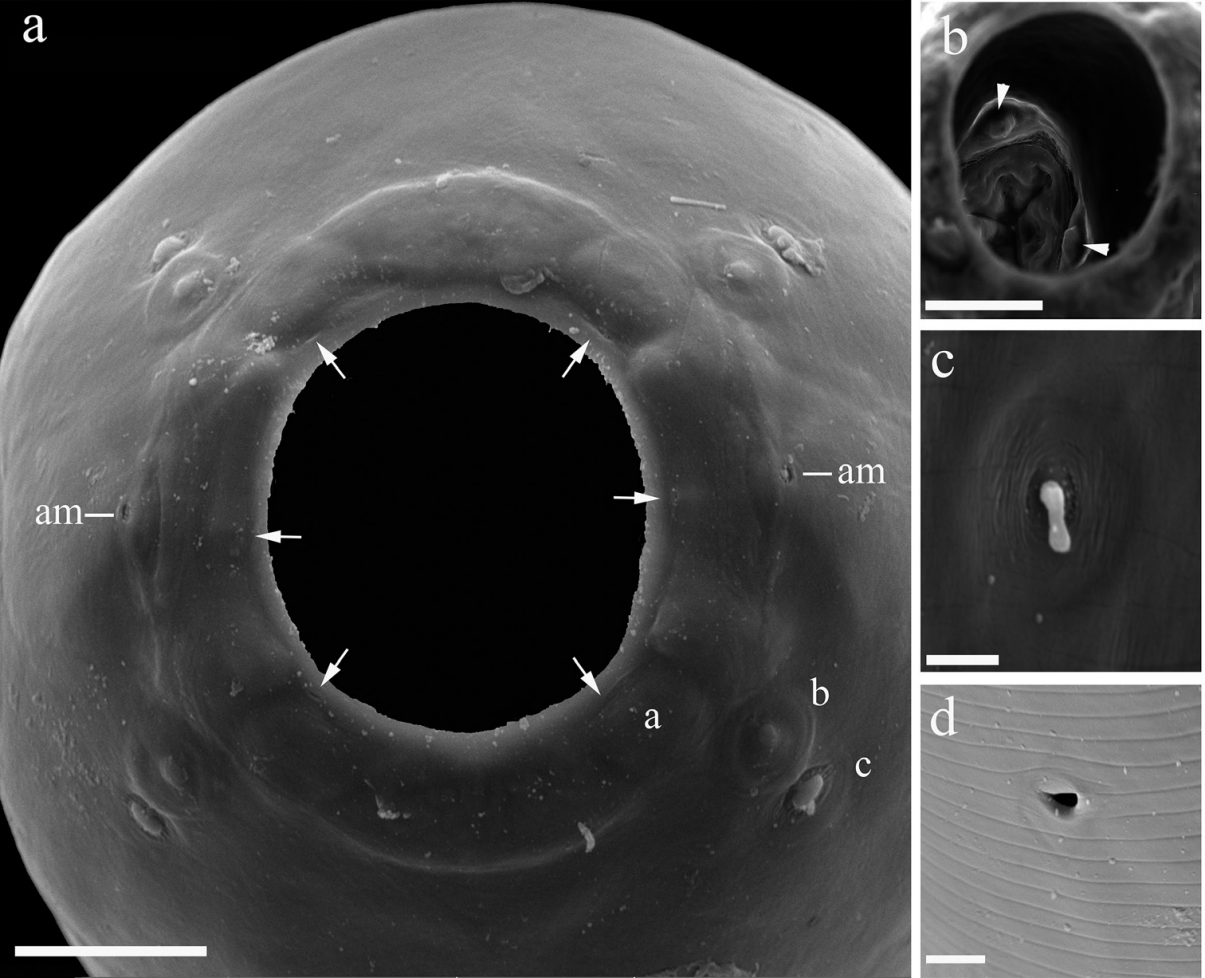
*Procamallanusspiculastriatus* sp. n., female, scanning electron microscopy. **a** oral opening, frontal view, three circles of cephalic papillae (a, b, c), amphid (am, arrowhead), pore-like structures (arrows) **b** oral opening, view of buccal capsule, two teeth are visible at the base of the basal ring (arrows) **c** deirid **d** excretory pore. Scale bars: 10 µm (**a, b**); 2 µm (**c**); 5 µm (**d**).

[Based on 10 males, 11 females, 20 eggs (from allotype female), and 20 intrauterine larvae (from allotype female)] Medium-sized nematodes, red while alive and white after fixation. Cuticle with fine transverse striations. Oral opening circular, surrounded by three concentric circles with four papillae each, inner circle with six small pores at base proximal to oral opening, pair of small lateral amphids present (Figure 2a). Buccal capsule, orange-brown, barrel-shaped with a well-developed basal ring armed with four sclerotized tooth-like structures (Figs 1a, b, 2b). Inner surface of capsule smooth (Figure 1a, b) without ridges. Muscular esophagus somewhat shorter than glandular esophagus. Deirids minute, simple with rounded tip, situated between the buccal capsule and nerve ring (Figure 2c).

**Figure 3.**
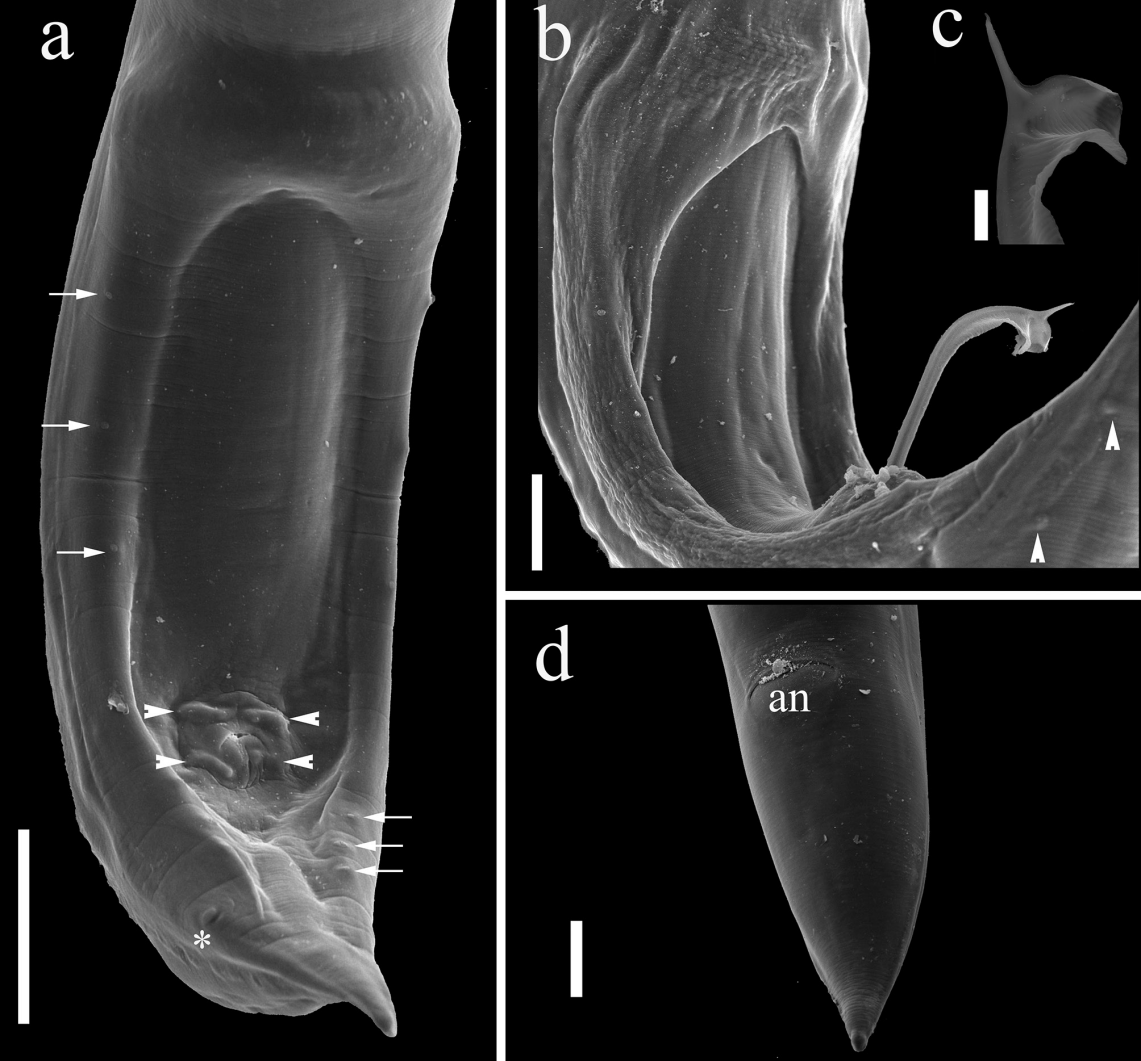
*Procamallanusspiculastriatus* sp. n., scanning electron microscopy. **a** tail of a male worm, ventral view, three preanal pairs (arrows) and three postanal pairs (arrows), four adcloacal papillae (arrowheads) and a lateral phasmid (*) are visible **b** tail of a male worm, lateral view with the spicule partially extroverted, and two pairs of sessile papillae are located along lateral margin (arrowhead); inset **c** detail of the tip of a spicule **d** tail of a female worm, ventral view, anus (an). Scale bars: 25 μm (**a**); 50 μm (**b**); 5 μm (**c**); 50 μm (**d**).

*Males (based on holotype and 9 paratypes)*: body 8–11 (9) mm long; maximum width at esophageal/intestinal junction 105–147 (130). Buccal capsule including basal ring 57–74 (65) long and 32–39 (36) wide, basal ring 5–8 (6.5) long, 22–29 (26) wide. Maximum length/width ratio of buccal capsule 1:0.55. Deirids, nerve-ring and excretory pore at 91–119 (104), 156–188 (171) and 248–292 (263), respectively, from anterior extremity. Muscular portion of esophagus 316–395 (353) long and 42–53 (48) wide; glandular portion of esophagus 421–558 (470) long and 42–63 (51) wide. Muscular/glandular esophagus length ratio 1:1.3. Length of entire esophagus and buccal capsule 9–12% of body length. Posterior end of body ventrally curved, provided with wide caudal alae bearing six pairs of pedunculated papillae: three precloacal pairs and three postcloacal pairs (Figs 1e, 3a). Two pairs of adcloacal papillae. (Figs 1e, 3a). Two pairs of dorso-lateral sessile papillae, between cloaca and tip of tail present (Figure 3b). Caudal alae anteriorly interconnected, forming a pseudosucker, and not reaching tip of tail; a pair of phasmids located immediately posterior to the 6^th^ pair of pedunculated papillae (Figs 1e, 3a). Spicules elongate and ventrally curved, with slightly sclerotized core; distal end of spicules with membranous alae supported by sclerotized rays (Figs 1e–h, 3b, c). Spicules with terminal bifurcation, similar in form, bifurcation of right spicule always larger than that of left spicule. Spicules dissimilar in length; left spicule shorter, 229–284 (247) long and right spicule 312–355 (332) long (Figure 1e–h). Gubernaculum absent. Length of tail 156–205 (184).

*Females with larvae (based on 4 specimens)*: body 17–20 mm (18 mm) long; maximum width at esophageal/intestinal junction 160–173 (167). Buccal capsule including basal ring 78–83 (81) long and 47–52 (50) wide, basal ring 12–13 (12) long, 32–43 (36) wide. Maximum length/width ratio of buccal capsule 1:0.62. Deirids, nerve-ring, and excretory pore at 147–167 (156), 220–233 (225), and 330–397 (371), respectively, from the anterior extremity (Figure 1c). Muscular portion of esophagus 413–453 (433) long and 53–60 (58) wide; glandular portion of esophagus 600–693 (633) long and 67–80 (70) wide. Muscular/glandular esophagus length ratio 1:1.5. Length of entire esophagus and buccal capsule representing 6–7% of body length. Vulva situated at, 8–11 (9) mm from anterior end, at about 50% of body length; vulval lips not elevated. Muscular vagina directed posteriorly (Figure 1c); uterus filled with larvae 210–280 (245±22 long) (Figure 1i) and eggs 17–29 (24) long by 14–26 (22) wide. Tail conical, 190–220 (208) long, without cuticular projections (Figs 1d, 3d).

*Females with eggs (based on 7 specimens)*: body 11–13 mm (12 mm) long; maximum width at esophageal/intestinal junction 100–133 (124). Buccal capsule including basal ring 80–87 (82) long and 50–60 (53) wide, basal ring 10–12 (10) long, 33–38 (35) wide. Maximum length/width ratio of buccal capsule 1:0.64. Deirids, nerve-ring, and excretory pore at 127–163 (144), 183–227 (208), and 283–357 (308), respectively, from the anterior extremity. Muscular portion of esophagus 387–460 (427) long and 47–60 (52) wide; glandular portion of esophagus 447–527 (489) long and 60–67 (61) wide. Muscular/glandular esophagus length ratio 1:1.5. Length of entire esophagus and buccal capsule representing 8–9% of body length. Vulva situated at 6–7 mm (6 mm) from anterior end, at 52% of body length; vulval lips not elevated. Muscular vagina directed posteriorly; uterus filled with eggs 28–32 (29) long by 25–30 (26) wide. Tail conical, 140–180 (166) long, without cuticular projections.

## Discussion

The family Camallanidae was established for species with a prominent, sclerotized buccal capsule (Railliet and Henry 1915). Yeh (1960) divided the family into two subfamilies, Camallaninae Railliet & Henry, 1915, for species with the buccal capsule divided into two halves, and Procamallaninae Yeh, 1960, for those with a single, cup-like buccal capsule. The new species with its cup-like buccal capsule composed of two lateral halves is identified as a member of Procamallaninae. Six genera have been assigned to Procamallaninae: *Procamallanus*, *Spirocamallanus* Olsen, 1952, *Malayocamallanus* Jothy & Fernando, 1970, *Punctocamallanus* Moravec & Scholz, 1991, *Spirocamallanoides* Moravec & Sey, 1988 and *Denticamallanus* Moravec & Thatcher, 1997 (Baylis 1923; Olsen 1952; Jothy and Fernando 1970; Moravec and Scholz 1991; Moravec and Thatcher 1997; Rigby and Rigby 2014). The genus *Procamallanus* consists of approximately 40 known species distributed worldwide, but only two species have been reported from Brazil, *P.peraccuratus* and *P.annipetterae* Kohn & Fernandes, 1988, both from southern Brazil (Pinto et al. 1976; Petter and Dlouhy 1985; Kohn and Fernandes 1988). The new species is assigned to *Procamallanus* because its cup-like buccal capsule with smooth walls (without striations); according to Moravec and Thatcher (1997) the main characteristic of this genus is the presence of a smooth buccal capsule in both sexes.

The new species can be distinguished from all known members of the genus outside of Brazil in having tooth-like structures on the basal ring of the buccal capsule. In addition to the above characteristic, it differs from *P.annulatus* Yamaguti, 1955 (Indonesia), *P.elatensis* Fusco & Overstreet, 1979 (Israel), *P.laeviconchus* Wedl, 1861 (Egypt), *P.planoratus* Kulkarni, 1935 (India) and *P.pseudolaeviconchus* Moravec & Van As, 2015 (Botswana) by the absence of a sclerotized gubernaculum, present in the other five species (Wedl 1861; Kulkarni 1935; Yamaguti 1955; Fusco and Overstreet 1979; Moravec and Van As 2015). *P.spiculastriatus* can be distinguished from *P.pacificus* Moravec, Justine, Würtz, Taraschewski & Sasal, 2006 (New Caledonia) also not found in Brazil, by the absence of the small processes (mucrons) (*sensu*Moravec et al. 2006) on the tip of the tail.

Two species of *Procamallanus* have been found in Brazil: *P.peraccuratus* Pinto, Fábio, Noronha & Rolas, 1976, from *Geophagusbrasiliensis* (Quoy & Gaimard) and *Australoherosfacetus* (Jenyns) (both Cichlidae) in the State of Espirito Santo (Southern Region of Brazil) and *P.annipetterae* Kohn & Fernandes, 1988 (= *P.petterae* Kohn & Fernandes, 1988), from *Corydoraspaleatus* (Jenyns) in the Iguaçu River, State of Paraná (south of Brazil) (Pinto et al. 1976; Kohn and Fernandes 1988). Pinto et al. (1976) suggested that the characteristics of the buccal capsule and the morphological and morphometric data of each taxon should be considered for the differentiation of species, a view shared by Moravec (1998).

*Procamallanusspiculastriatus* sp. n. has tooth-like structures on the basal ring of the buccal capsule similar to these in *P.annipetterae* although the new species has four distinct tooth-like structures, whereas these are six in *P.annipetterae* as described by Petter (1990; see also her fig. 5A) in addition the letter species is distinct. Kohn and Fernandes (1988) provide no info on the number of cephalic papillae in females; only in the holotype male *P.spiculastriatus* sp. n. can be distinguished from *P.annipetterae* by the tail shape (conical in female of the new species vs. pointed in both sexes with a marked long and narrow posterior part); number of caudal papillae (three precloacal, two adcloacal, and five postcloacal vs. four precloacal and four postcloacal), shape of oral opening (circular in *P.spiculastriatus* vs. oval in *P.annipetterae*) and morphometric parameters such as spicule length (smaller spicule 229–284 µm, larger spicule 312–355 µm vs. 150–160 µm and 180–210 µm, respectively) and length of the tail (184 µm in males and 208 µm in females vs. 336 µm in males and 281 µm in females), comparisons made based on the description of Kohn and Fernandes (1988).

*Procamallanusspiculastriatus* sp. n. resembles *P.peraccuratus* in the morphology of the buccal capsule, oral opening circular and presence of caudal alae of males, but differs by the presence of four internal sclerotized tooth-like structures on the basal ring, the presence of two postcloacal dorsal papillae, and the presence of spicules with alate distal end supported by sclerotized rays of *P.spiculastriatus* and those characters are absent in *P.peraccuratus* (Moravec et al. 1993; Moravec 1998). Despite sharing hosts from the same family (Cichlidae), the species differ with respect to species of host and the biomes where they are found: *P.spiculastriatus* sp. n. is a parasite of *A.ocellatus* (biome Amazonia), whereas *P.peraccuratus* is a parasite of *G.brasiliensis* and *Au.facetus* (biome Brazilian Atlantic Forest). This finding extends the geographical distribution of the genus into the Brazilian Amazon. Additional morphometric comparisons between *P.spiculastriatus* sp. n. and the two species found in Brazil are presented in Table 1.

**Table 1.** Comparison of morphometric characteristics of the known South American species of *Procamallanus* with those of *Procamallanusspiculastriatus* n. sp. Except as noted for individual characteristics, all data for *P.peraccuratus*, and *P.annipetterae* were taken from the original descriptions.

**Caracteres**	***Procamallanusspiculastriatus* n. sp.**				*** P. peraccuratus ***		*** P. annipetterae ***	
	**Holotype**	**Allotype**	**Male**	**Female**	**Male**	**Female**	**Male**	**Female**
Length (mm)	10.76	17	8–11	17–20	9.42–9.75	12.78–22.34	9.69	21.8
Width	147.36	166	105–147	160–173	150–170	210–400	500	720
Buccal capsule (L)a	64.93	80	57–74	78–83	72–87	87–113	131	180
Buccal capsule (W)	38.96	51	32–39	47–52	49	52–66	123	187
Mouth opening	Circular		Circular		Circular		–	
Teeth	Present	Present	Present	Present	Absent	Absent	Present	Present
Deirids	107.79	150	91–119	147–167	–	–	–	–
Nerve ring	168.83	226.66	156–188	220–233	220	230–240	298	326
Excretory pore	280.51	330	248–292	330–397	–	260–330	–	–
Muscular esophagus (L)	352.63	420	316–395	413–453	410–440	560–660	625	644
Glandular esophagus (L)	557.89	600	421–558	600–693	450–520	580–660	868	887
Ratio L/Oc and esophagus	9%	6.5%	9–12%	6–7%	10.32%^b^	7.57%^b^	16.76%^b^	7.8%^b^
Vulva (mm)	–	8	–	8–11	–	6.7–10.90	–	–
Preanal papillae (pairs)	3	–	3	–	3	–	2	–
Additional papillae (pairs)	2	–	2	–	2	–	2	–
Postanal papillae (pairs)	3 + 2DL^a^	–	3 + 2DL^a^	–	4	–	1	–
Spicule large	342.17	–	312–355	–	270-290	–	210	–
Spicule small	230.8	–	229–284	–	180–200	–	160	–
Caudal alae	Present	–	Present	–	Present	–	Absent	–
Tail	166.23	190	156–205	190–220	140	220–310	336	281
Host	* Astronotus ocellatus *				*Geophagusbrasiliensis* and *Australoherosfacetus*		* Corydoras paleatus *	
Site	Intestine				Intestine		Intestine	
Locality	Belém, Pará, Brazil				Vale do Rio Itaúnas, Espírito Santo, Brazil		Rio Iguaçu, Paraná, Brazil	
Biome	Amazonia				Atlantic Forest		Atlantic Forest	
Author	In this study				Pinto et al. (1976)		Kohn and Fernandes (1988)	

^a^Abbreviations: L = length; W = width, e = esophagus, DL = dorsolateral papillae; Ratio L/Oc and esophagus = Length of entire oesophagus and buccal capsule representing of body length. ^b^Derived or calculated from the original publication of the species description.

The six genera currently assigned to Procamallaninae were reduced to subgenera of *Procamallanus* by Moravec (1998). However, Moravec (1998) did not provide any arguments why the former genera should be demoted to a lower level. Rigby and Rigby (2014) noted the overlap in the diagnostic characteristics of the buccal capsules of these taxa. Two molecular phylogenetic studies (Černotíková et al. 2011; Choudhury and Nadler 2016), further revealed that *Procamallanus* and *Spirocamallanus* are paraphyletic. We find that phylogenetic relationships within Procamallaninae have not been evaluated in an objective analysis using both morphological and molecular data. There is no evidence that such different taxa should be assigned to the same genus-level group (i.e., that they should be recognized as sub-genera rather than distinct genera, which is an arbitrary decision). Therefore, based on the current knowledge of the studied group we follow the concept accepting the full generic status of all recognized genus-group names within Procamallaninae. Even in the absence of a rigorous test of the monophyly of the genera, all but *Procamallanus* have contain species with consistent features; all known species of *Procamallanus* have smooth-walled buccal capsules without tooth-like structures except for *P.spiculastriatus* sp. n. and *P.annipetterae*, both of which have tooth-like structures on the basal ring. This suggests that these two species might represent a distinct genus. Currently, we do not feel confident in describing a new genus without the support of a formal phylogenetic analysis. Among the camallanid species parasitizing *A.ocellatus*, only *Spirocamallanusinopinatus* from northern Brazil (Moravec 1998; Thatcher 2006) and *Camallanus* sp. from Midwestern Brazil have been reported (Kohn et al. 1985; Vicente et al. 1985).

## Supplementary Material

XML Treatment for Procamallanus spiculastriatus
